# RIEMS: a software pipeline for sensitive and comprehensive taxonomic classification of reads from metagenomics datasets

**DOI:** 10.1186/s12859-015-0503-6

**Published:** 2015-03-03

**Authors:** Matthias Scheuch, Dirk Höper, Martin Beer

**Affiliations:** Institute of Diagnostic Virology, Friedrich-Loeffler-Institut, Federal Research Institute for Animal Health, Südufer 10, 17493 Greifswald - Insel Riems, Germany

**Keywords:** Metagenomics, Data analysis, Taxonomic classification

## Abstract

**Background:**

Fuelled by the advent and subsequent development of next generation sequencing technologies, metagenomics became a powerful tool for the analysis of microbial communities both scientifically and diagnostically. The biggest challenge is the extraction of relevant information from the huge sequence datasets generated for metagenomics studies. Although a plethora of tools are available, data analysis is still a bottleneck.

**Results:**

To overcome the bottleneck of data analysis, we developed an automated computational workflow called RIEMS – *R*eliable *I*nformation *E*xtraction from *M*etagenomic *S*equence datasets. RIEMS assigns every individual read sequence within a dataset taxonomically by cascading different sequence analyses with decreasing stringency of the assignments using various software applications. After completion of the analyses, the results are summarised in a clearly structured result protocol organised taxonomically. The high accuracy and performance of RIEMS analyses were proven in comparison with other tools for metagenomics data analysis using simulated sequencing read datasets.

**Conclusions:**

RIEMS has the potential to fill the gap that still exists with regard to data analysis for metagenomics studies. The usefulness and power of RIEMS for the analysis of genuine sequencing datasets was demonstrated with an early version of RIEMS in 2011 when it was used to detect the orthobunyavirus sequences leading to the discovery of Schmallenberg virus.

**Electronic supplementary material:**

The online version of this article (doi:10.1186/s12859-015-0503-6) contains supplementary material, which is available to authorized users.

## Background

Chen and Pachter [1] defined the analysis of metagenomes as “the application of modern genomics techniques to the study of communities of microbial organisms directly in their natural environments, bypassing the need for isolation and lab cultivation of individual species”. Metagenomic applications are significantly supported by the various next generation sequencing (NGS) technologies by reducing the cost per base while simultaneously raising both the throughput and the output. All sequencers feature a massive parallelisation in raw sequence generation mainly differing in their sample preparation and sequence detection method as well as in the required run time. All these technologies enable researchers and diagnosticians to answer various questions ranging from de novo sequencing, whole genome or target-region resequencing, transcriptome research, RNA sequencing and epigenomics to metagenomics [2]. The application of NGS enables unbiased and comprehensive sequencing of genomic material from diverse samples, regardless of origin and composition. Therefore, the generated sequence reads, i.e. short sequences representing individual fragments of the input nucleic acid molecules reflect the composition of the original sample material both qualitatively (with regard to the comprised species) and quantitatively (with regard to the abundances of the detected species). In addition to qualitative and quantitative analyses, the sequences allow functional classification, i.e. classification with regard to the functions predicted for any detected sequence. The aforementioned possibilities make metagenomics a powerful tool both scientifically and diagnostically. Diverse scientific applications of metagenomics are reviewed in [3,4]. Scientifically, as mentioned before, the analysis of the microbial community with regard to its taxonomic composition and metabolic capabilities is of interest. Diagnostic metagenomics may be applied if a causative agent of a disease is suspected but cannot be detected by targeted diagnostics. In this case, the agent may be identified based on sequence similarities of even only a single read to known sequences with a relation to database-listed pathogen genomes. Hence, diagnostic metagenomics primarily aims at the taxonomic classification of the sequences. For instance, Palacios and co-workers [5] identified a novel virus with similarity to an old world arenavirus as the causative agent in a study of three patients who died of a febrile illness 4 to 6 weeks after receiving organ transplantations from the same donor. Another prime example for the diagnostic application of metagenomics was the detection of Schmallenberg virus (SBV). Here, the detection of only 7 reads with similarity to orthobunyavirus sequences within a dataset of roughly 27,500 reads provided the necessary information for diagnosis, virus isolation, and finally experimental proof of SBV as the causative agent of disease [6].

As outlined above, metagenomics has the potential to answer diverse questions. However, analyses of the huge datasets that are produced in metagenomics studies require enormous computing capacity for the analysis in order to generate and extract information from the raw sequence data. Meanwhile, complete workflows exist to analyse metagenomic sequence data, for example MG-RAST [7] and the EBI metagenomics service [8] (https://www.ebi.ac.uk/metagenomics/). Moreover, comprehensive lists of tools for the different analysis parts are available [3,9-11]. The workflows apply different strategies for initial data preparation to ensure efficient analyses. These strategies include initial clustering [12], mapping along reference sequences [13,14], or assembling reads into contigs (sets of overlapping reads) [15,16]. After initial data preparation, the sequences are classified taxonomically or functionally, again applying different strategies using various tools.

For taxonomic classification, diverse software applications were designed which all use different algorithms but rely on the analysis of 16S rRNA sequences. Software enabling the comparison of datasets obtained from different habitats is inter alia DOTUR [17]. This tool clusters 16S rRNA sequences into operational taxonomic units (OTUs) or phylotypes and analyses genetic distances between sequences. The principle of DOTUR was expanded in the software SONS [18]. The application LIBSHUFF [19] compares samples by statistical comparison of their 16S rRNA sequence contents. The tool UniFrac [20] calculates evolutionary distances of multiple environments based on phylogenetic information and multivariate statistical techniques. All these applications are limited to 16S rRNA sequences, hence only prokaryotic sequences can be analysed. In addition to the different aforementioned types of exclusively taxonomic classifications, the tool Qiime [21] allows the combination of OTU based taxonomic classification with additional analyses. These additional analyses include sequence alignments, inference of phylogenetic trees, and phylogenetic or taxon-based analysis of diversity. Like Qiime, PhyloPythia [22] also enables phylogenetic analyses. It uses a multiclass support vector machine classifier with the oligonucleotide composition of variable-length genome fragments for the generation of phylogenetic sequence clades. For the analysis of read sequences, PhyloPythia is rather inappropriate due to the fact that its accuracy decreases dramatically by using input sequences of length less than 1,000 nucleotides [22] as is the case for reads.

Besides the rRNA based taxonomic classification of the sequence reads, the reads can also be classified both taxonomically and functionally based on similarity searches at the nucleic acid or the amino acid level. Analyses at the nucleic acid sequence level are e.g. performed with BLAST, HMMer, or BLAT [23-25]. Glimmer, FragGeneScan, or GeneMark [26-28] perform assessments of open reading frames using probabilistic models in order to rate the coding capacity of the raw sequences. All the aforementioned applications generate only per read results which must subsequently be further processed to extract and summarize the relevant information. Thereto, the software MEGAN [29] can be used to explore the content of a complete metagenomic dataset. MEGAN also provides results of the analysis as graphical and statistical output thereby enabling comparison of different datasets.

A limitation of all taxonomic and functional binning tools is the analysis duration due to the linear correlation between analysis time and data size. To speed up the binning, hardware improvements are possible. Another way is to split the query data into subsets for parallel analysis in a network of multiple servers like it is realised in cloud computing [30] and with the software MetaGeniE [31]. The throughput of similarity based binning can also be increased by using fast heuristic approaches applied for instance in the UCLUST and USEARCH algorithms [32]. However, application of UCLUST and USEARCH is restricted by the memory requirements for the analysis which proportionally increases ten times with the size of the database. The performance problem can be circumvented by using publicly available web services like MG RAST [7] and the EBI metagenomics analysis service [8]. Certainly, their capacity is limited as well, leading to considerably long duration of the analyses. Therefore, it might be more effective to run the analysis using locally installed applications. Moreover, in certain settings, for instance when dealing with confidential diagnostic data, it might be necessary to have a local analysis running. This is for instance possible with the recently released software SURPI [33]. After pre-processing the reads, SURPI first screens the query dataset for human sequences followed in fast mode by classification of the reads as viral or bacterial based on reference datasets for viral and bacterial sequences, respectively. Only in comprehensive mode, SURPI screens the pre-processed reads against the complete NCBI nt database and then assembles the reads class-wise to proceed with proteome analysis based on assembled sequences. Other software for local installation also include Readscan [34] and RINS [35], both of which require prior knowledge in form of user input reference sequences the dataset has to be screened for. In case of RINS, the reads are initially mapped to a user provided custom query dataset thereafter filtered for uniqueness and then screened for human sequences. Finally, the non-human reads are assembled and the resulting contigs are classified using blast. Since reads that do not map to the user provided query are exempted from the analysis, the user will only detect what he is explicitly looking for as defined by the query dataset. Similarly, in the case of Readscan, it is necessary to provide datasets classified as host and pathogen references, respectively. The reads are aligned along these sequences and according to the highest percent identity with a reference sequence the reads are finally binned into the predefined classes. Therefore, also in case of Readscan the user needs prior knowledge to provide suitable pathogen and host reference datasets. The crucial importance of the design of reference datasets for the successful correct classification is stressed by the authors of Clinical PathoScope [36]. Likewise, the authors of Kraken [37] emphasize the importance of a validated dataset to be used as the basis of the database in use. Clinical PathoScope, like Kraken [37] and MetaPhlAn [38], can be run locally on a unix server from the command line and produce a comprehensive result summary.

Taken together, although a plethora of different applications and workflows for the analyses of metagenomics data is available, there are various limitations of these. Most importantly, the analyses take too long, the tools do not cover full taxonomic content (e.g. rely on rRNA and therefore will not detect viruses), are by default centred on human sample analysis, or they do not generate a comprehensive and clear result protocol (e.g. BLAST) for further use. Of course, these limitations are due to the specialized aims of the tools. Nevertheless, the resulting obstacles need to be cleared away. Therefore, here we present RIEMS (*R*eliable *I*nformation *E*xtraction from *M*etagenomic *S*equence datasets), our software workflow for sensitive and reliable analysis of metagenomic datasets. Unlike a number of tools mentioned above, which by default assume human sample origin for host screening, RIEMS by default neither requires prior information concerning the sample origin nor other input for read classification, e.g. to distinguish between host and pathogen. Rather, RIEMS automatically detects the most abundant species and screens the complete dataset for the respective sequences. For this purpose, RIEMS combines several established software applications. Different sequence analyses are cascaded with decreasing stringency of the assignments to allow for the highest possible reliability and sensitivity of the read classifications. While the above mentioned workflows act linearly on the complete input datasets, i.e. screening out host reads followed by a single round of read assignments, RIEMS repetitively runs through detection of abundant species. To this end, RIEMS repeatedly extracts random data subsets which are assembled and classified and subsequently the complete dataset is screened for the detected species. Moreover, for unbiased taxonomic classification based on the most similar sequence, RIEMS does not rely on restricted databases but uses the full content of the INSDC databases. As an additional analysis layer, RIEMS can switch to amino acid sequences for the analyses of reads and contigs remaining taxonomically unclassified after analysis of the nucleic acid sequences. Finally, all assignments are summarised in diverse output files as well as in a clearly structured result protocol, classified taxonomically. An early version of RIEMS proved the power of its approach in 2011 when it was used to detect the orthobunyavirus sequences leading to the discovery of Schmallenberg virus [6].

## Results and discussion

As outlined above, the aim of the presented work was to provide a tool for the automated sensitive and reliable taxonomic classification of all individual reads comprised in metagenomics sequence datasets. The flow of the analyses performed by RIEMS including a rationale for the combination and order of the different software tools is described in detail in the Methods section. The most important output of RIEMS is a tabular summary of the classifications plus the sorted reads for further use and additional information regarding the different analyses that were carried out.

### RIEMS output

The output of RIEMS is stored per input dataset in a separate folder containing a number of result files and subfolders reflecting the different parts of the analysis. In the main folder, the original input file, a progress report and a result protocol listing all identified organisms can be found. Within the folders for the various analysis parts, separate result files, lists of read accessions which were assigned to taxa during the respective analyses and files summarizing alignment information of BLAST analyses are stored.

**Figure 1:**
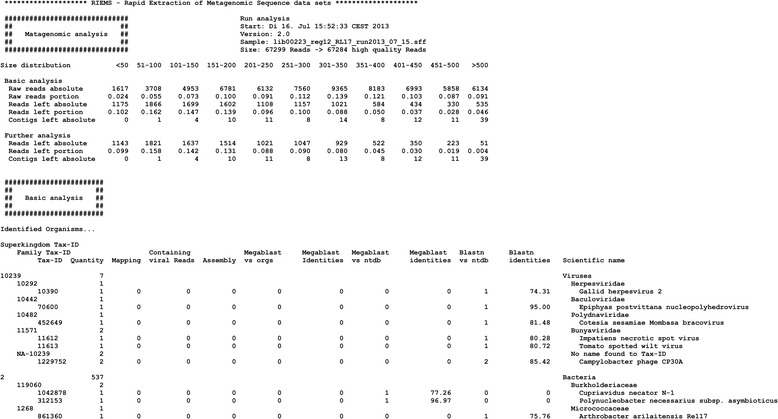
**Cut-out of a result protocol of the ‘Basic analysis’.** The first lines show general information concerning the analysis, followed by a read size distribution calculated before and after the ‘Basic analysis’ as well as calculated after the ‘Further analysis’. The lower part displays the number of reads assigned to the detected species grouped at the domain and family levels. The central columns individually represent the results for mapping, assembly, and BLAST showing the number of reads detected by the respective tool. Blast results are accompanied by the range of identities of the read with the best hit in the database.

The most important output of RIEMS is the tabular summary of all results. This is stored in both tab-separated values (tsv) and plain text format. This protocol is divided into three sections, first a paragraph of general information, thereafter a summary of organisms identified during the ‘basic analysis’, and finally a synopsis of those identified in the further analysis. The top of Figure 1 shows the general information of the result protocol which contains the current version of RIEMS, start date and time of the analysis, the input file name, and the total number of reads in the input file together with the number of reads that passed the initial quality filtering. The next section is a summary showing the read length distribution of the original dataset, the reads remaining unclassified after ‘basic analysis’ and ‘further analysis’, respectively, as well as those that finally remained unclassified.

The second section of the result protocol (Figure 1) is the summary of organisms identified within the read sequences during the ‘Basic analysis’. This section is structured according to the taxonomy at the levels of “superkingdom”, “family”, and “species” with one taxonomic entity per line. The leading columns contain the taxonomic information given as the taxonomy IDs from the NCBI taxonomy database, the last column shows the scientific name of the entity. For every entity, the total number of reads assigned to this entity and besides this information, a breakdown of the numbers of reads assigned by the different combinations of tools and databases is given. For all BLAST results, the protocol contains information of the sequence identities of the query, i.e. the read sequence, and the best hit. If more than one read is classified into that taxonomic entity by the respective BLAST, the sequence identities are given as the range of the lowest and the highest identity. Additionally, the number of reads that potentially are viral sequences assigned to a eukaryote by the mapping is indicated in a dedicated column. The second section ends with a summary of the numbers of reads and contigs that could not be assigned during the ‘basic analysis’.

**Figure 2:**
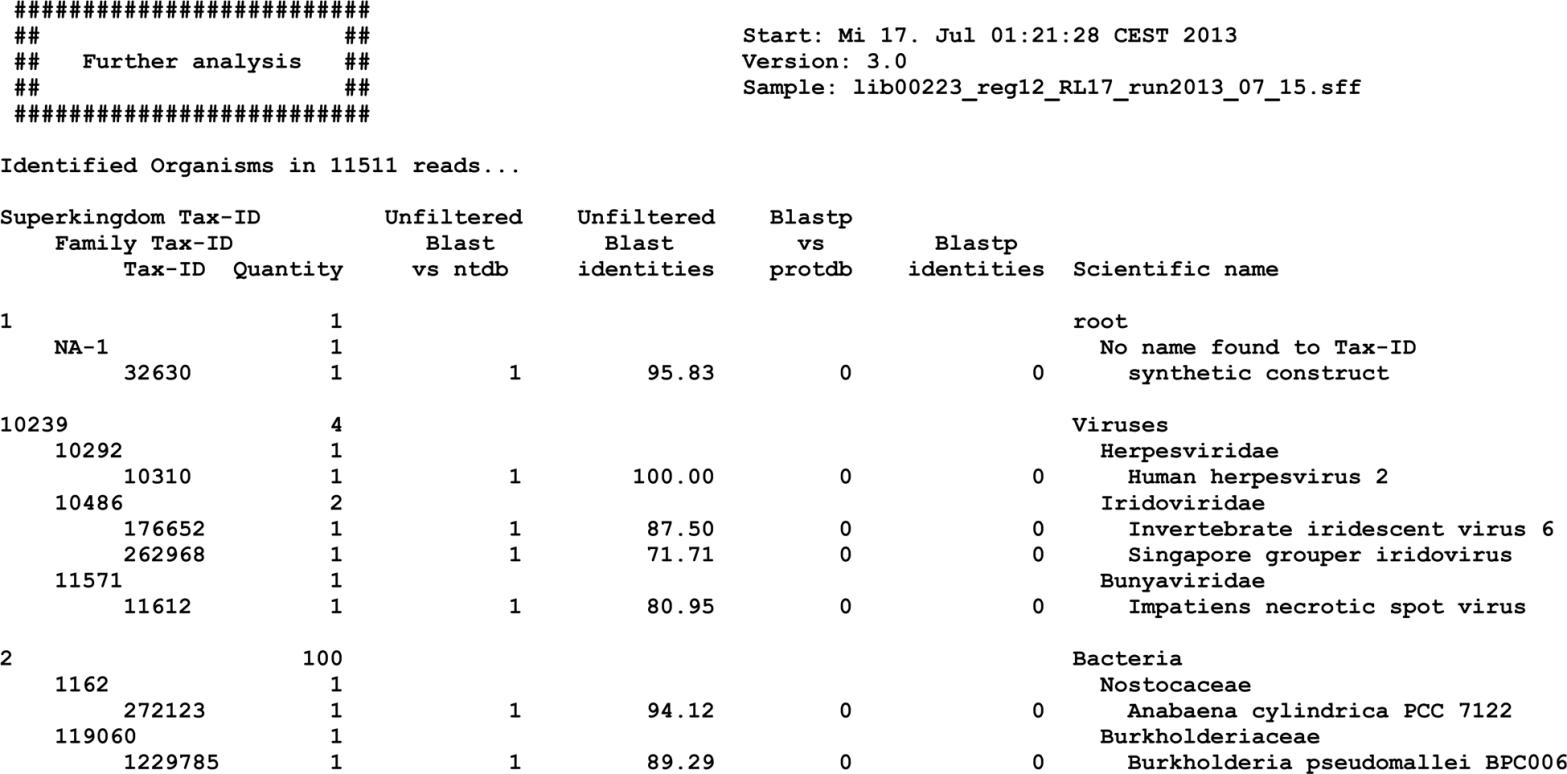
**Cut-out of a result protocol of the ‘Further analysis’ for reads.** The first lines show general information of date, version, sample, and the number of reads used as input for the analysis. The following table displays the number of reads assigned to the detected species structured at the domain and family levels. The central columns individually display the number of reads detected by the different BLAST analyses. Blast results are accompanied by the range of identities of the read with the best hit in the database.

**Figure 3:**
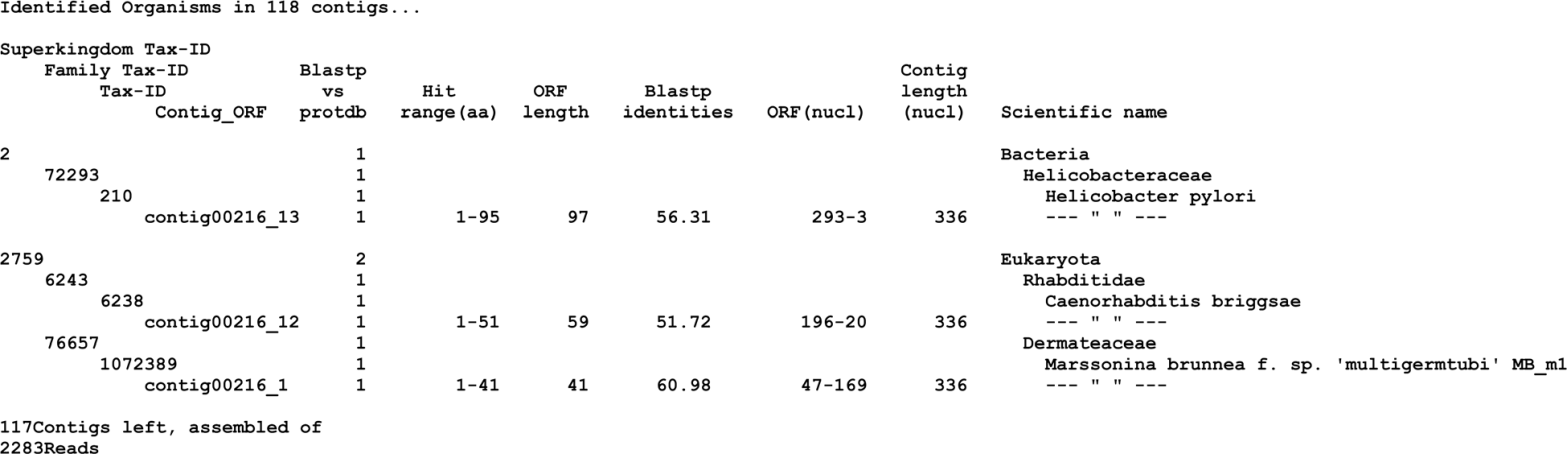
**Cut-out of a result protocol of the ‘Further analysis’ for contigs.** The first line shows the number of contigs analysed. The following table displays the BLASTp hits for ORFs deduced from the nucleotide sequences of contigs that could not be taxonomically classified by nucleotide sequence analyses. The ORFs are assigned to species and the results are structured according to taxonomic domain and family levels. Additional columns show information about the deduced aa sequence and the alignment.

The third section of the result protocol contains information of the ‘Further analysis’, again the results are structured taxonomically with an identical representation as in section 2 (‘Basic analysis’). Section 3 is subdivided in two parts (Figures 2 and 3). The first part of section 3 (Figure 2) is a summary of the nucleotide blast analyses (both megablast and blastn) without the low complexity filter. Besides the nucleotide blast results, the results of the blastp analyses of the amino acid sequences deduced from reads is summarized. Like in the second section (results of the ‘Basic analysis’), the identities of the query with the hit sequences are given. Part two of section 3 (Figure 3) displays the assignments of ORFs detected in the contigs that were assembled during the ‘Basic analysis’ but remained unassigned. For the contigs, the table lists information about the assigned ORF in addition to the numbers of sequences assigned to the respective taxon and the sequence identities. The ORF information encompasses the ORF identifier, the aa range that was identified, the identity of the identified aa sequence with the subject sequence, the ORF length (codons), the position of the ORF within the contig, and the length of the contig. Part 2 of section 3 ends with a list of the total number of unassigned reads and contigs plus the number of reads assembled into these contigs. Below the results, a short description of the protocol and the finishing date and time are listed.

### Validation and evaluation of RIEMS

RIEMS was tested with respect to its accuracy and robustness of the assignments and with respect to its performance, i.e. the time necessary for complete analysis, using genuine 454 sequencing as well as simulated sequencing datasets. The simulated datasets were composed of (partial) genome sequences of six bacterial species, three viruses, and two eukaryotes (Table 1). These genomes were split into overlapping fragments with length distributions equal to those from 454 pyrosequencing. The resulting approximately 90,000 simulated reads were combined into a single dataset. In addition, we used a simulated dataset from the Clinical PathoScope project (http://sourceforge.net/projects/PathoScope) for validation. This dataset comprises 10,000,000 reads of each 100 nucleotides length representing human, bacterial and viral sequences.

**Table 1:** **Detailed information of sequences used to generate the simulated sequencing dataset for validation and comparison**

**Domain**	**Sequence description**	**Genbank identifier**	**Accession**
Bacteria	*Bacillus anthracis* str. CDC 684 chromosome	227812678:c1-285375	NC_012581.1
	*Escherichia coli* O104:H4 str. 2011C-3493 chromosome	407479587:c1-457939	NC_018658.1
	*Burkholderia mallei* SAVP1 chromosome I	121598179:c1-586553	NC_008785.1
	*Clostridium botulinum* BKT015925 chromosome	331268188:c1-461407	NC_015425.1
	*Staphylococcus aureus* 08BA02176 chromosome	404477334:c1-460546	NC_018608.1
	*Yersinia pestis* A1122 chromosome	384137007:c1-457660	NC_017168.1
Viruses	Akabane virus segment M	157939617:c1-4309	NC_009895.1
	Newcastle disease virus isolate 2009_Mali_ML008	355467763:c1-15027	JF966387.1
	Influenza A virus (A/muscovy duck/Vietnam/LBM295/2012(H5N1)) viral cRNA, segment 7, complete sequence	464101994:c1-992	AB807883.1
Eukaryota	*Bos taurus* DNA sequence from clone CH240-405I18	445065096:c1-177923	FO393397.2
	*Canis lupus* familiaris clone rp81-289 m11	34787525:c1-193729	AC129099.6

#### Validation

The accuracy of RIEMS was first assessed using our simulated sequencing dataset. All species originally represented in the dataset were detected. The assignments of reads representing eukaryotes (10,255 reads) and viruses (538 reads) were carried out with 100% specificity and close to 100% sensitivity (Table 2). Assignments of reads generated from bacterial sequences (79,592 reads) showed a slight decrease with regard to sensitivity (median 98.7%, minimum 96.6%, maximum 100%) and specificity (99.1% to 100%; Table 2). Here, additional species were identified (see Additional file 1: Table S1). This is caused by highly homologous sequences found in different bacterial species due to direct relations of the species and/or horizontal gene transfer. Assessment of the accuracy using the Clinical PathoScope dataset resulted in similar figures (Table 3). In this case, we used the pre-screening feature of RIEMS to screen the sample for reads representing human sequences. The specificity of the classifications was 100% except for human sequences (99.1%), the median sensitivity was determined as 97.8% (minimum 15%, maximum 99.7%; Table 3). The minimum sensitivity was reached for Influenza A virus where 83% of the reads were classified as synthetic construct, all representing synthetic Influenza A virus constructs, nevertheless not counted as correctly identified.

**Table 2:** **Results of RIEMS validation using our simulated sample dataset with original (upper half) and deviating (lower half) sequences**

**Input species**	**True positive**	**False positive**	**True negative**	**False negative**	**Unclassified**	**Sensitivity**	**Specificity**	**Positive predictive value**	**Negative predicitive value**	**Correct classification rate**	**False classification rate**
**Original sequences**											
*Bacillus anthracis*	12774	731	76880	0	0	100	99.06	94.59	100	99.19	0.81
*Burkholderia mallei*	16092	102	74129	62	0	99.62	99.86	99.37	99.92	99.82	0.18
*Clostridium botulinum*	12300	0	77649	436	0	96.58	100	100	99.44	99.52	0.48
*Staphylococcus aureus*	12425	0	77678	282	0	97.78	100	100	99.64	99.69	0.31
*Escherichia coli*	12289	0	77761	335	0	97.35	100	100	99.57	99.63	0.37
*Yersinia pestis*	12551	0	77788	46	0	99.63	100	100	99.94	99.95	0.05
*Newcastle disease virus*	418	0	89967	0	0	100	100	100	100	100	0
*Akabane virus*	119	0	90266	0	0	100	100	100	100	100	0
*Influenza A virus*	1	0	90384	0	0	100	100	100	100	100	0
*Bos taurus*	4906	0	85476	3	2	99.94	100	100	100	100	0
*Canis lupus*	5346	0	85039	0	0	100	100	100	100	100	0
**Deviating sequences**											
*Bacillus anthracis*	12762	633	76978	12	0	99.91	99.18	95.27	99.98	99.29	0.71
*Burkholderia mallei*	16055	79	74152	99	0	99.39	99.89	99.51	99.87	99.8	0.2
*Clostridium botulinum*	12358	1	77648	378	0	97.03	100	99.99	99.52	99.58	0.42
*Staphylococcus aureus*	12484	1	77677	223	0	98.25	100	99.99	99.71	99.75	0.25
*Escherichia coli*	12400	0	77761	224	1	98.23	100	100	99.71	99.75	0.25
*Yersinia pestis*	12529	0	77788	68	1	99.46	100	100	99.91	99.92	0.08
*Newcastle disease virus*	418	0	89967	0	0	100	100	100	100	100	0
*Akabane virus*	119	0	90266	0	0	100	100	100	100	100	0
*Influenza A virus*	1	0	90384	0	0	100	100	100	100	100	0
*Bos taurus*	4887	0	85476	22	5	99.55	100	100	99.97	99.98	0.02
*Canis lupus*	5346	0	85039	0	0	100	100	100	100	100	0

**Table 3:** **Results of RIEMS validation using the Clinical PathoScope simulated sample dataset**

**Input species**	**True positive**	**False positive**	**True negative**	**False negative**	**Unclassified**	**Sensitivity**	**Specificity**	**Positive predictive value**	**Negative predicitive value**	**Correct classification rate**	**False classification rate**
*Haemophilus influenzae*	344646	17	9643530	7010	3	98.01	100	100	99.93	99.93	0.07
*Homo sapiens*	8975152	8541	986662	24848	7	99.72	99.14	99.9	97.54	99.67	0.33
*Human mastadenovirus B*	59289	0	9931563	4351	0	93.16	100	100	99.96	99.96	0.04
*Human bocavirus*	9	0	9995193	1	0	90	100	100	100	100	0
*Human coronavirus NL63*	24375	0	9970203	625	5	97.5	100	100	99.99	99.99	0.01
*Enterovirus A*	911	0	9994203	89	0	91.1	100	100	100	100	0
*Human respiratory syncytial virus*	9855	0	9985203	145	56	98.55	100	100	100	100	0
*Rhinovirus C*	241	0	9994953	9	0	96.4	100	100	100	100	0
*Influenza A virus*	15	0	9995103	85	0	15	100	100	100	100	0
*Moraxella catarrhalis*	169607	7	9823264	2325	1	98.65	100	100	99.98	99.98	0.02
*Streptococcus pneumoniae*	193919	18	9800120	1146	0	99.41	100	99.99	99.99	99.99	0.01
*Streptococcus intermedius*	175739	47	9818606	811	0	99.54	100	99.97	99.99	99.99	0.01

Furthermore, we evaluated the robustness of RIEMS with the simulated sequencing dataset to simulate a situation in which novel species are comprised in the sample. To this end, the Emboss tool ‘msbar’ [39] was used to introduce five random mutations per read (insertions, deletions, substitutions), resulting in an error rate between 1% and 10%, depending on the read length. A total of 90,378 of the modified artificial reads were assigned and only 7 reads (<0.008%) remained unassigned. As for the original simulated sequencing dataset, all species originally comprised were identified correctly despite the introduced deviations. Again, some additional species were detected (Additional file 1: Table S1). Altogether, the modified sequences were assigned with a median specificity of 100% (minimum specificity 99.2%; Table 2) and a median sensitivity of 99.6%. Like for the original sequences, the sensitivity was higher for eukaryotic and viral sequences (median 100%, minimum 99.6%; Table 2) than for the bacterial sequences (median 98.8%, minimum 97.0%, maximum 99.9%; Table 2). Hence, neither the sensitivity nor the specificity of RIEMS classification are significantly compromised by deviations from known related sequences.

#### Performance

**Figure 4:**
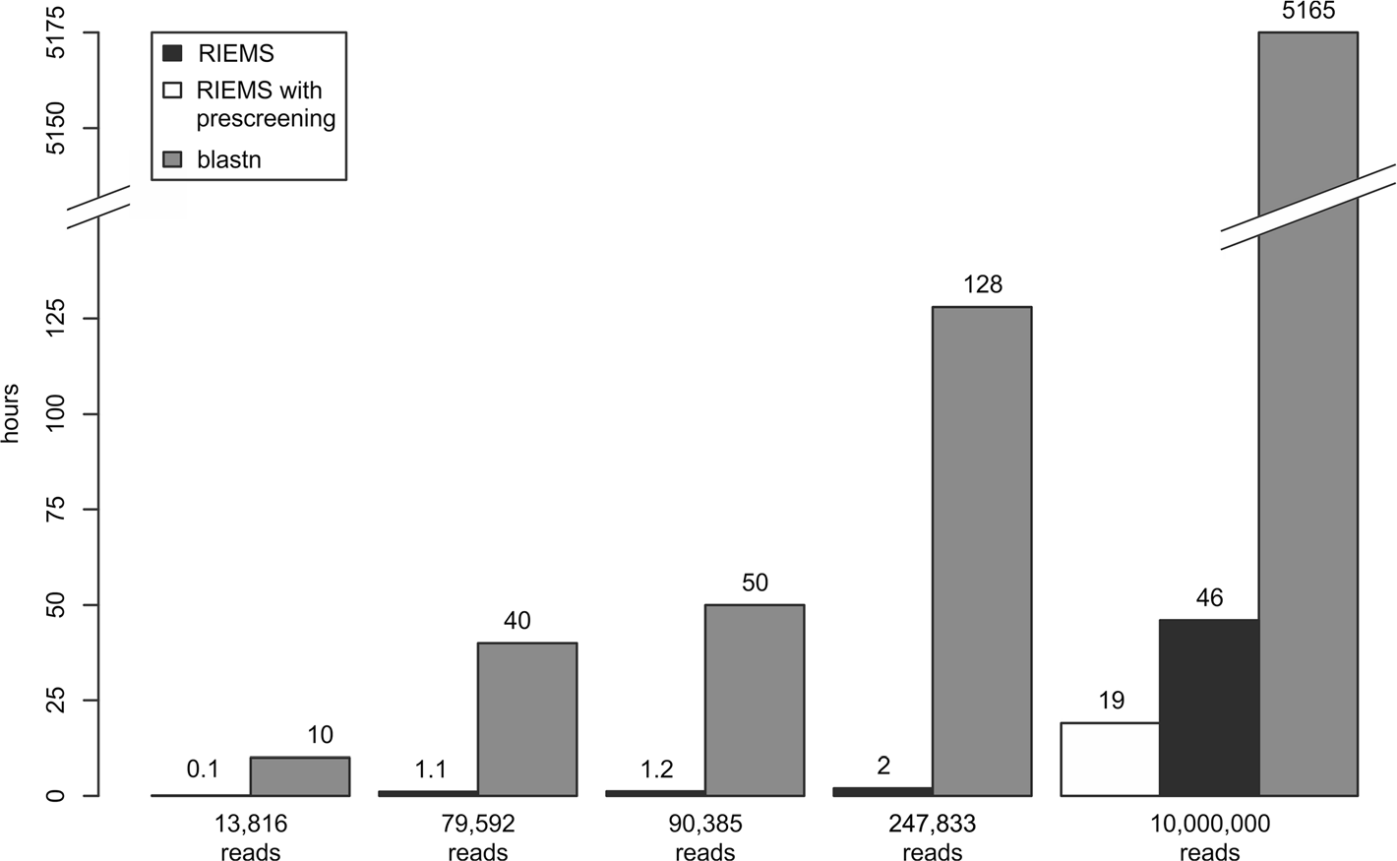
**Comparison of the analysis duration of RIEMS and blastn analysis.** All computations were performed locally using 24 cores and the NCBI nt database. For all datasets, RIEMS classified 99.9% of all reads. For the larger datasets, the duration of blastn analysis was extrapolated from the dataset with 13,816 reads.

In order to determine the efficiency of RIEMS, different datasets were analysed both using RIEMS and as a benchmark blastn [23] because the classifications of both are nearly identical. Both analyses were run on the same server with identical conditions using the maximum number of available cores (n = 24). Five different datasets were used for benchmarking; three of these were simulated sequencing datasets comprising 79,592, 90,385, and 10,000,000 reads, respectively, and two genuine sequencing datasets (13,816 reads; 247,833 reads). The simulated datasets were the same that were used for the validation. The results of the comparison are shown in Figure 4. While RIEMS classified 99.9% of the 13,816 genuine reads within only 6 minutes, blastn analysis [23] (NCBI nt database) of the same dataset required approximately 10 hours. Extending the dataset roughly linearly increases the duration of blastn analyses due to the approximately linear correlation between the number of sequences and blastn duration. Therefore, the time necessary for the blastn analyses of the larger datasets was extrapolated. Unlike blastn, an increase of the sequence volume does not necessarily cause an increase of the time necessary to analyse the data using RIEMS. RIEMS analyses of 79,592; 90,385; 247,833; and 10,000,000 reads, respectively, always classified ≥99.9% of the reads and took between one and two hours, except for the Clinical PathoScope simulated sample dataset (with default settings 46 hours; by performing an optional pre-screening for human sequences, the duration could be reduced to approximately 19 hours; Figure 4). In conclusion, using RIEMS, there is no direct correlation between the number of sequences and the analysis time, but the duration mainly depends on the complexity of the dataset.

#### Comparison with other software

RIEMS was compared with web-based workflows and other software running locally, all representing different concepts of the program structure and different classification algorithms for the analysis of metagenomic datasets. A major reason for inclusion in the comparison was that the software like RIEMS does not require user provided prior knowledge. Moreover, since the crucial importance of validated reference datasets has been stressed [36,37], we restricted the comparisons to tools for which the necessary validated databases are provided. Namely, we used the web-services MG-RAST [7] and EBI metagenomics [8] to analyse the simulated sequencing dataset of 90,385 reads and the programs Kraken [37], Clinical PathoScope [36], and the MetaPhlAn clade specific marker database [38] for the analysis of our simulated dataset and the Clinical PathoScope simulated dataset.

Comparison of RIEMS and the 2 web-services was hampered by the fact that these do not provide classifications per read but only overall figures. For both MG-RAST and EBI Metagenomics only the simulated read dataset of 90,385 original reads was used. The MG-RAST [7] results were formatted to resemble the RIEMS results as far as possible (assignments were limited to the best hit; annotation source set to ‘GenBank’ with a maximum e-value cut-off of 10^−4^; minimum percentage identity cut-off 60%; minimum alignment length cut-off 15; result table grouped according to families). MG-RAST classified nearly 100% of the reads with an overall accuracy of approximately 90% at the read to family level. Except the *Canidae*, all families comprised in the dataset were detected but only a few hundreds instead of thousands of reads were assigned to the *Bovidae*. However, MG-RAST assigned more reads than actually present in the dataset to bacterial families except the *Bacillaceae*. In contrast, too few reads were assigned to the virus families *Paramyxoviridae* and *Bunyaviridae* by MG-RAST. Furthermore, 4,701 simulated sequencing reads were assigned to 130 families not being part of the original data. For a more detailed comparison of the RIEMS and the MG-RAST results please refer to Additional file 1: Table S2. For the second comparison, the simulated sequencing dataset of 90,385 reads was analysed by the EBI metagenomics service [8]. Comparison of the results of RIEMS and EBI metagenomics is hampered by the different classification strategies and data output structures. Most notably, RIEMS does not perform any functional analysis like EBI metagenomics does. On the contrary, EBI metagenomics taxonomic analyses are performed by QIIME [21] only based on the detected 16S rRNA sequences. Hence, EBI metagenomics did not identify a single viral sequence comprised in the dataset. In order to enable the comparison of the results, 488 16S rRNA sequences comprised in the original dataset of 90,385 reads were selected and analysed using RIEMS. Subsequently, the assignments of reads to OTUs by EBI metagenomics or to taxonomic classes by RIEMS were compared (Table 4). RIEMS classified all reads except one correctly; the only falsely classified read belonging to the bacilli was classified to the betaproteobacteria. EBI metagenomics [8], i.e. QIIME [21], also assigned the majority of the clostridial and betaproteobacterial reads to the correct OTUs (deviation less than 1%). However, for the bacillal sequences, with a deviation of 47% the accuracy of the OTU assignments was rather low. These reads were grouped to the alphaproteobacteria, unknown firmicutes, unknown proteobacteria, or unknown bacteria (Table 4).

**Table 4:** **Comparison of RIEMS and EBI metagenomics results**

			**Assignments by**					
	**16S rRNA reads comprised**		**RIEMS**			**EBI Metagenomics**		
**Class**	**absolute**	**%**	**Reads detected**		**Deviation (basis point)**	**OTUs detected**		**Deviation (basis point)**
			**absolute**	**%**		**absolute**	**%**	
Bacilli	387	79.3	386	79.1	0.2	27	33.3	46.0
Clostridia	51	10.5	51	10.5	0.0	8	9.9	0.6
Unknown firmicutes	-	-	-	-	-	2	2.5	2.5
Alphaproteobacteria	-	-	-	-	-	3	3.7	3.7
Betaproteobacteria	50	10.3	51	10.5	0.2	8	9.9	0.4
Unknown proteobacteria	-	-	-	-	-	2	2.5	2.5
Unknown bacteria	-	-	-	-	-	30	37.0	37.0
Assigned			488	100		80	98.7	
Unassigned	-		0	0.0		1	1.3	
Total	488	100.0	488	100		81	100	

For this comparison a data subset of 488 16S rRNA sequences comprised in the simulated sequencing dataset was used. The deviation is presented in basis point between the percentage of the respective assignments and the percentage in the original dataset.

**Figure 5:**
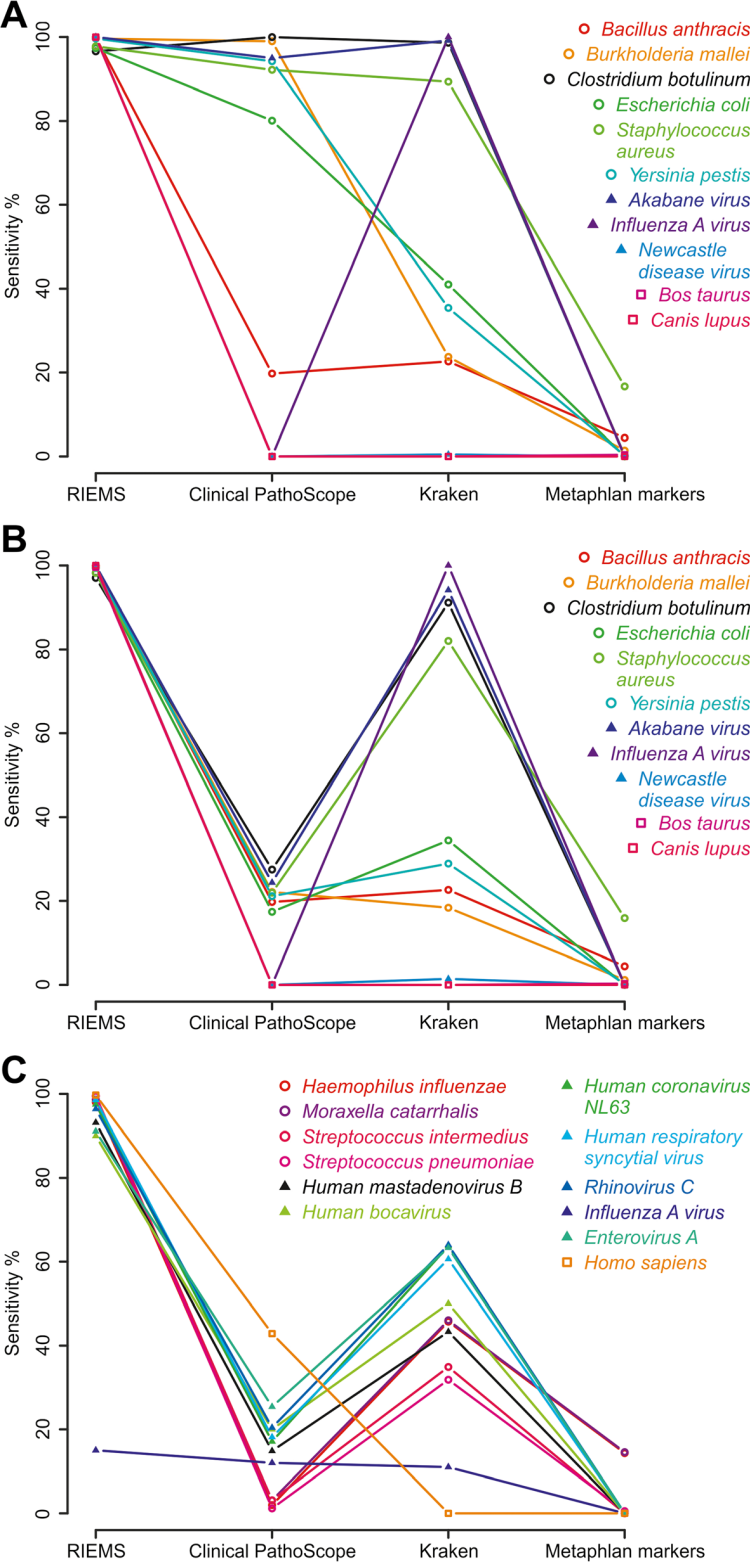
**Comparison of the sensitivities of RIEMS, Clinical PathoScope, Kraken, and Megablast against the MetaPhlAn clade specific marker database.** The plots show the sensitivities calculated from the read to species assignments using the three simulated sample datasets. **(A)** Our simulated sample comprising 90,385 reads representing original sequences derived from viral, bacterial, and eukaryotic genome sequences (see Table 1). **(B)** The same dataset as used in **(A)** but with 5 deviations per read. **(C)** The Clinical PathoScope simulated sample dataset.

The analyses run locally using Kraken, Clinical PathoScope, and the MetaPhlAn approaches are substantially faster (analyses duration in the range of minutes) than RIEMS (hours) (see Additional file 1: Table S3). All tools detected all species targeted by the prebuilt validated databases provided by the respective authors. Based on the read to species assignments, all 4 programs had specificities close to 100% for all analysed species they target. However, the results for both simulated datasets revealed substantial differences in the sensitivities of the different software in combination with the provided databases (Figure 5) for the different species comprised in the datasets. These differences are most likely caused by the databases we used since restricted databases generally cause a reduced sensitivity [31]. Additionally, our results imply that a restriction of the analysis to only bacteria or bacteria plus viruses poses a problem because a huge proportion of the reads may be left unclassified and hence no information may be available whether these reads represent host sequences or novel microorganisms leaving a substantial portion of uncertainty. Noteworthy, the sensitivity of Clinical PathoScope substantially decreased when analysing the dataset comprising sequences deviating between 1% and 10% from the original sequences (compare Figures 5A and B). Therefore, in the case of novel sequences with too low similarity with the chosen references these novel sequences will not be classified.

To test the suitability of the different tools in combination with the databases provided by the authors for the classification of novel sequences, we constructed a dataset of 2,827 genuine Illumina MiSeq reads (FASTQ formatted) representing 2 novel viruses from our unpublished sequencing projects. Only reads were chosen that were previously assembled into the respective genomes and could unambiguously be identified as viral sequences. The chosen viruses are not yet classified by the International Committee on Taxonomy of Viruses but additional experimental evidence exists regarding their taxonomic classification. Their genome sequences are not available in the sequence databases and viruses and sequences will be published elsewhere. These reads were analysed with Kraken, Clinical PathoScope, and RIEMS; the MetaPhlAn clade specific marker database was not used because this only targets bacteria but not viruses. Using the NCBI nt database (as of 30.09.2014) which does not comprise the respective sequences, RIEMS classified all 2,827 reads correctly (read to species classification) at the nucleotide sequence level. Additional analyses at the aa level were not necessary. On the contrary, both Kraken and Clinical PathoScope classified none of the reads at all.

#### Analysis of genuine sequencing datasets

Throughout the development of RIEMS, the workflow was used to analyse experimental and diagnostic datasets. In all cases, the relevant information from these datasets could be extracted and used for the identification of viruses, for complete genome assemblies, or as references to set up specific real-time RT-PCR assays.

In order to compare RIEMS with other analysis strategies, datasets from two published studies were re-analysed. First, raw data from a study in which a new avian bornavirus (ABV) was identified [40] were analysed. This dataset consisted of approximately 121,000 reads. RIEMS required less than 1.5 hours for the detection of reads and contigs representing the new ABV variant. Here, based on the deduced amino acid sequences the ‘Further analysis’ unambiguously identified the genome as a viral sequence belonging to the species *avian bornavirus* within the family *bornaviridae* although the identity of the newly detected with known ABV genome sequences was too low to be identified by nucleotide sequence analyses. Secondly, data from a study of Sachsenröder and co-workers [41] were analysed. The authors of this study identified a pig stool-associated circular ssDNA virus (PigSCV). RIEMS analysis of this second dataset (approx. 70,000 reads) gained equivalent results with regard to the identified organisms and viruses including the new PigSCV. Also in combination with sample preparation specifically targeting sequencing to viral nucleic acids, data analysis using RIEMS proved to be useful and powerful [42].

Most importantly, RIEMS analysis of sequences generated from diagnostic samples from cows with undiagnosed disease and unspecific symptoms yielded seven orthobunyavirus reads with low identity to Akabane virus sequences amongst ca. 27,000 reads within one hour. These reads provided the initial hint for the identification of the novel Schmallenberg virus [6]. The sequences of the identified viral reads were easily retrieved from the complete dataset and were used for the development of a specific real-time RT-PCR assay. Using this RT-qPCR assay, the detected virus was confirmed as the causative agent and the spread of the virus could be determined.

## Conclusions

While sequencing capacities are steadily increasing and simultaneously the price per base is decreasing, there is a substantial lack in powerful tools for efficient data analysis. Meanwhile, the bottleneck of exploiting the full potential of NGS is determined by data analysis capacities. Especially the analysis of metagenomics datasets is complex and time consuming. Although various tools and strategies were published and are publicly accessible, the available capacities are not sufficient. Moreover, the tool of choice depends on the aim of the study. All existing tools and pipelines have their strengths and shortcomings. A general issue seems to be incorrect classification of reads to species. All software programs tested in this study did classify reads incorrectly. This is an intrinsic problem of sequence classification caused by high degrees of identity between the genomes of different strains and even between different related species. However, the specificity of the read classifications is not the major problem but rather the sensitivity seems to be an issue. The reduced sensitivity in a number of settings for the analysis of unbiased metagenomics sequence data is caused by the fact that they don’t do unbiased data analysis due to restricted reference databases. This causes a substantial loss in sensitivity especially for the classification of sequences with only limited similarity to known sequences. Here, RIEMS has the potential to fill the gap that still exists. All comparisons showed a high sensitivity for RIEMS analyses and at the same time a high specificity. With its only very limited software dependencies RIEMS is suitable for local installation, thereby minimizing the need to transfer huge datasets via the internet and helping to maintain data confidentiality. Moreover, the output of RIEMS is clearly structured taxonomically and the sorted sequence reads can easily be used for successive analyses. This allows the integration of RIEMS analysis into existing workflows, for instance sorting the raw data prior to assembly. Of course, the combination of RIEMS with various other tools for metagenomics analyses is possible, enabling the quick and comprehensive characterization of metagenomics datasets.

## Methods

### Implementation, integrated software and databases

RIEMS is implemented as a unix shellscript written in bourne-again shell (bash). The script files are available under the GNU General Public License Version 3 together with the validation datasets and the “novel viruses dataset” at http://www.fli.bund.de/no_cache/en/startseite/institutes/institute-of-diagnostic-virology/labs-working-groups/laboratory-for-ngs-and-microarray-diagnostics.html. RIEMS only relies on validated standard software and databases. All software applications are installed on a local server (specifications see below). RIEMS has access to the 454 genome sequencer software suite (works with version 2.6 and later; Roche, Mannheim, Germany; available free of charge after registration at http://454.com/contact-us/software-request.asp), in particular the assembler Newbler and the GS reference mapper as well as the standard flowgram format (sff) and the FASTA nucleic acid (fna) tool commands. Furthermore, the NCBI BLAST software suite (version 2.2.26+; available at ftp://ftp.ncbi.nlm.nih.gov/blast/executables/blast+/) and the NCBI databases (available at ftp://ftp.ncbi.nlm.nih.gov/blast/db/) for nucleotide (nt), protein (nr), and taxonomy are integrated [23]. In addition, the Emboss software package (version 6.3.1; http://emboss.sourceforge.net/download/) [39] is included, in particular the applications for open reading frame (ORF) detection and amino acid translation. All software applications are used with default settings. The BLAST hit selection is based on the e-value (cut-off 0.001); the hit with the lowest e-value is selected. RIEMS accepts sequences input in the common sequence formats sff (as generated by Lifetechnologies IonTorrent and 454/Roche Genome Sequencers), FASTQ (for instance Illumina), or FASTA; in case of FASTA files preferably in combination with the corresponding quality files.

### Hardware and operating system

RIEMS and all necessary applications are installed locally on a server equipped with four Intel Xeon E7450 processors (each 6 cores with a frequency of 2400 MHz) and 64 GB PC2-5300 (DDR2-667 MHz) ECC RAM. The server is linked with 2 × 4 Gbit/s ports to the storage area network. The operating system is CentOS release 5.10 with Linux kernel release 2.6.18-371.1.2.e15.

### Delineation of RIEMS data processing

**Figure 6:**
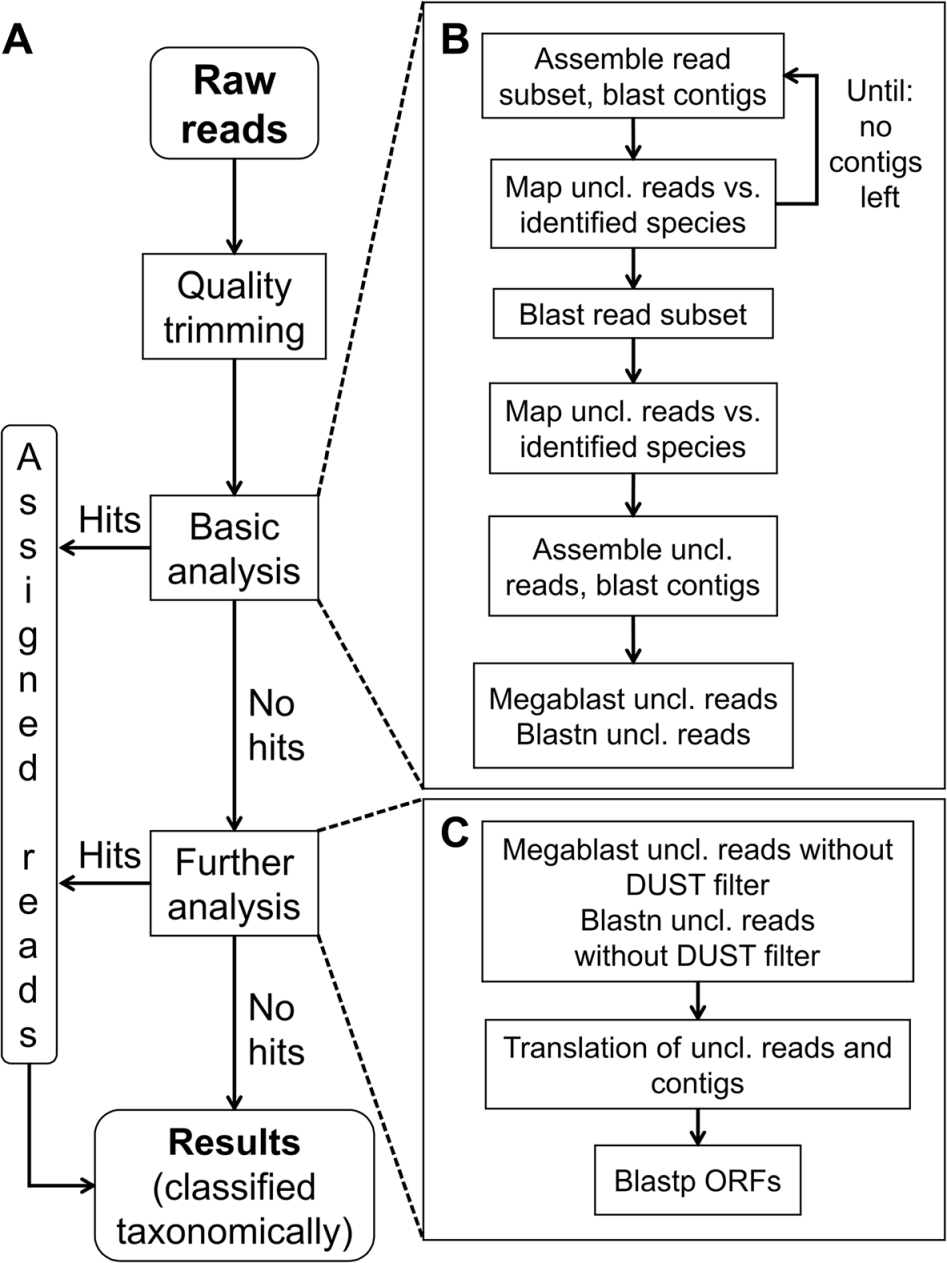
**Flow diagram of RIEMS. (A)** Main steps of RIEMS analysis. **(B)** Succession of analyses within the ‘Basic analysis’ **(C)** Principal steps of the ‘Further analysis’. For details see text.

A general overview of the RIEMS pipeline is depicted in Figure 6. RIEMS is subdivided into a ‘Basic analysis’ which taxonomically assigns the bulk of the dataset and a ‘Further analysis’ dealing with all sequences remaining taxonomically unassigned after the ‘Basic analysis’. Both the basic and the further analysis follow the general strategy of sequentially applying different tools with decreasing stringency in order to achieve a fast and reliable overall analysis. After both the basic and the further analysis are finished, all results are summarized in a spread sheet arranged taxonomically.

Prior to all analyses, all reads are quality trimmed for high reliability of the final classifications. For this purpose, the GS reference mapper application is used which intrinsically performs a quality trimming. Thereto, a poly-A sequence of 120 nucleotides in length is generated and used as reference for a mapping (Figure 6). The trim points which are determined during this quick initial mapping are then used as fixed trim points in all analyses. In parallel, a pre-screening of the dataset against a user provided reference set can optionally be performed like shown in the analyses of the Clinical PathoScope dataset (Figure 4).

#### Basic analysis

The main steps of the ‘Basic analysis’ (Figure 6B) are identification of the sample background, assignment of reads to the background species, identification of further abundant species in the remaining dataset and a final blast analysis with decreasing stringency, first using megablast followed by blastn.

The first step of the actual analysis is the identification of the sample background, i.e. the species the bulk of the reads can be assigned to. For this purpose, a subset of 5,000 reads is randomly extracted from the trimmed reads and assembled into contigs using the 454 newbler assembler. Subsequently, the resulting contigs are analysed using megablast against the NCBI nucleotide database “nt” (all GenBank + EMBL + DDBJ + PDB sequences) [23]. Based on GenBank identifiers (GIs) of the best hits, the associated taxonomy IDs of the corresponding species are determined in the NCBI taxonomy database. This information is used to retrieve all sequences of each detected species from the nucleotide database. Successively, each of these sequence sets is first used as reference for a mapping and thereafter a megablast [23] analysis in which reads excluded from the mapping due to length restriction or partially mapped reads are included (a detailed depiction of the repetitive taxonomy based sequence retrieval and alignment procedure can be found in Additional file 1: Figures S1, S2, and S3). In case an identified organism is a eukaryote, all reads that were classified to this organism by mapping are blasted against a partial database containing only viral sequences in order to identify viral reads potentially classified as eukaryotic reads due to viral sequences which may be part of eukaryotic genomes (Additional file 1: Figure S3). All reads that were classified in one of these steps are exempted from subsequent analyses. This procedure (generating and assembling a random sequence subset, followed by BLAST, sequence retrieval and mapping plus scanning for viral sequences) is repeated with the remaining unassigned reads until either no new species are identified by blasting the generated contigs or no new contigs can be assembled. This repeated procedure enables a rapid, automatic breakdown of the dataset without user provided knowledge of the organisms making up the sample background.

If after the initial repeated background analyses more than 10,000 reads are still unassigned and cannot be assembled into contigs, another random sub-setting process is initiated. However, in this process, only 100 reads are taken from the remaining unassigned reads and are analysed directly using megablast [23] without a prior assembly. The taxonomy IDs of the hits are determined according to their GenBank Identifiers (GIs) obtained from the BLAST result. Only if more than eight reads are assigned to the same taxonomy ID the corresponding sequences are retrieved from the nucleotide database and used as reference for a mapping and a subsequent megablast analysis like in the initial background analysis (Additional file 1: Figures S2 and S3).

All reads that remain unassigned after the initial screening are assembled into contigs. Reads that remained unassembled in this step are assigned to the contigs by megablast if at least 80% of the bases can be aligned to the contig. Subsequently, all contigs are searched against the local nucleotide database using blastn [23]. If BLAST aligns contigs only partially and the unaligned part consists of more than 30 consecutive nucleotides, this unaligned part is again searched in the database by blastn. Based on the assignment of a contig to an organism, reads that were aligned to that contig by megablast are assigned to the respective organism as well (Additional file 1: Figure S4). Unassigned and partially assigned contigs are saved in a file for the further analysis (see below).

Those reads that were neither assembled into a contig nor assigned to a contig by megablast in the previous assembly step are sequentially investigated by multiple BLAST analyses. The first of these BLAST analyses is a megablast against a database encompassing the nucleotide sequences of all organisms that were identified in the partial datasets and used as references for mappings. Secondly, the remaining unassigned reads are searched in the NCBI nt database again using megablast. Finally, blastn is used to search the unaligned remainder in the NCBI nt database. In all of these BLAST searches, unaligned parts of more than 30 consecutive nucleotides of partially aligned reads are searched a second time (Additional file 1: Figure S5).

#### Further analysis

The ‘Further analysis’ can optionally be executed and starts with additional nucleotide BLAST analyses of the reads. Then, for the subsequent analyses the sequence type is switched to the amino acid sequences coded by the sequences that remained unassigned (reads and contigs). Due to the higher information content of the 20 letter amino acid alphabet, usually additional sequences can be classified.

The initial nucleotide read sequence analyses within the ‘Further analysis’ are performed without low complexity filtering first using megablast and subsequently blastn both searching in the NCBI nt database. Deactivation of the low complexity filter further decreases the stringency of the assignments because even low complexity regions of the sequences are considered (Additional file 1: Figure S6). Hence, there is the chance that additional reads can be classified.

The next step is the translation of the unclassified reads (Emboss sixpack [39]) and contigs (Emboss getorf [39]) into amino acid sequences in all six frames. The only limitation we apply is a lower size cut-off of 20 amino acids because shorter peptide sequences cannot be analysed by blastp to yield a truly significant result; hence all ORFs with a minimum length of 20 codons are translated independently of start codons until reaching a stop codon. These settings assure that even in the case of sequencing noise causing non-sense mutations by insertions, deletions or single base exchanges the coded aa sequences can be analysed at least as partial sequences. In the case of short reads, i.e. Illumina HiSeq style reads, the lower size cut-off of 20 aa still permits their analysis. The deduced amino acid sequences are binned according to their length in order to select the proper substitution matrices for blastp according to [43,44] (<35 amino acids PAM-30, 35 – 50 amino acids PAM-70, 50 – 85 amino acids BLOSUM-80 and >85 amino acids BLOSUM-62). For contigs, all deduced aa sequences are analysed by blastp vs. NCBI nr database (Additional file 1: Figure S7). Blastp analyses (vs. NCBI nr database) of aa sequences deduced from reads (Additional file 1: Figure S8) start with the longest sequences translated from the reads, and only proceeds with the next shorter aa sequence deduced from that read if no hit was found in the database. If a similar aa sequence was detected all additional aa sequences for the respective read are exempted from further blast analyses.

### Comparison with other software tools

For the comparison of RIEMS with other tools with regard to sensitivity and specificity, we used Kraken [37] with the available Kraken Minidatabase (as of 30.09.2014), Clinical PathoScope [36] (v. 1.0.3, and database as of 30.09.2014) and the MetaPhlAn Clade Specific marker BLAST database [38] (as of 30.09.2014) in combination with Megablast for read classification; all three were used with default settings as recommended by the respective authors in combination with the validated databases provided at the project websites. For the comparisons we used different datasets, namely our simulated sample (see Table 1) with and without deviations, a small dataset comprising roughly 2,800 genuine Illumina MiSeq reads representing 2 different novel viruses, and the simulated sample #1 from the Clinical PathoScope project (http://sourceforge.net/projects/PathoScope). Here we had to modify the read accessions of reads representing Streptococcal sequences, because the authors didn’t label the reads according to the two different Streptococcal species present in the dataset. This was done by majority according to the classifications of the 4 tools in use. Roughly 4800 reads had to be discarded because of inconclusive classifications. Like RIEMS, the three tools put out read-wise classifications which were analysed using R (v3.1.0) [45] and R-Studio (v 0.98.507; RStudio, Inc. www.rstudio.com) to calculate the sensitivity and specificity. To enable a robust comparison of the different tools, classifications were consolidated to the species level and only unambiguous read-to-species classifications were taken into account. In case of Clinical PathoScope, reads longer than 100 bases are by default split into fragments; RIEMS has the capability to split reads if only partial matches occur. In both cases, results obtained for the sub-sequences were consolidated at the read level and again only counted if the results were unambiguous.

## Additional file

Additional file 1:
**Supplementary figures and tables.** The file contains the supplementary **Figures S1 – S8** and the respective figure legends and supplementary **Tables S1 – S3**.
