# Supplementary material for: RIEMS: a software pipeline for sensitive and comprehensive taxonomic classification of reads from metagenomics datasets
Source: BMC Bioinformatics. 2015 Mar 3;16(1):69. doi: 10.1186/s12859-015-0503-6 (PMC4351923; doi:10.1186/s12859-015-0503-6)
Supplement: Additional file 1: — Supplementary figures and tables. The file contains the supplementary Figures S1 – S8 and the respective figure legends and supplementary Tables S1 – S3. [file 12859_2015_503_MOESM1_ESM.docx]

**Supplementary Information for**

Scheuch et al.,

**RIEMS: A software pipeline for sensitive and comprehensive taxonomic classification of reads from metagenomics datasets**

**Icon legend**

- Input/output data

- Connection to the next element

- Operation;

- Conditional branch

- Subroutine

**Abbreviations**

Tax-ID - taxonomy id

Tax-ID Db - database generated of all taxonomy id sequences

# - number

eq. - equal

NtDb - INSDC nucleotide database

Prot Db - INSDC protein database

ORFs - open reading frames

aa - amino acids

sm - substitution matrix

**Supplementary figure 1** RIEMS ‘Basic analysis’; detailed flow diagram of the initial repetitive taxonomy based sequence retrieval and alignment procedure (part 1). For this part of the workflow, a subset of the reads is first assembled into contigs and these are used to identify comprised species. For further details, please refer to the main text.

**Supplementary figure 2** RIEMS ‘Basic analysis’; detailed flow diagram of the initial repetitive taxonomy based sequence retrieval and alignment procedure (part 2). For this second part of the workflow, a subset of the reads is used to identify comprised species. For further details, please refer to the main text.

**Supplementary figure 3** RIEMS ‘Basic analysis’; details of the subroutine ‘Mapping trimmed reads vs. Tax-ID sequences’ (cf. supplementary figures 1 and 2). All sequences related to an identified taxonomy id are successively retrieved from the database and used as a reference for a mapping and a megablast of the remaining unassigned reads. In case of eukaryotic taxonomy ids, a screening of the reads for viral sequences is performed. Further details can be found in the main text.

**Supplementary figure 4** RIEMS ‘Basic analysis’; schematic overview of the final assembly. All unclassified reads used as input for an assembly. All unassembled reads are blasted (megablast) against the generated contigs. Subsequently, contigs are taxonomically classified by BLAST analysis.

**Supplementary figure 5** RIEMS ‘Basic analysis’; overview of the final BLAST steps. All unassigned reads are first searched in a database comprising all available sequences of identified species. Thereafter, the still unclassified reads are searched in the nucleotide database using megablast and subsequently blastn.

**Supplementary figure 6** RIEMS ‘Further Analysis’; overview of the initial read sequence analyses. All reads that remained unassigned after the ‘Basic analysis’ are first analysed using megablast and then using blastn, both run without the low complexity filter.

**Supplementary figure 7** RIEMS ‘Further Analysis’; overview of the analyses of unassigned contigs from the ‘Basic Analysis’ final assembly. The contig sequences are translated to amino acid sequences and these are classified according to their length for use of the proper substitution matrix in the subsequent blastp analysis.

**Supplementary figure 8** RIEMS ‘Further Analysis’; overview of the final analyses of unassigned reads. The read sequences are translated to amino acid sequences and these are classified according to their length for use of the proper substitution matrix in the subsequent blastp analysis.

## Supplementary Table 1 - Overview of false classifications by RIEMS using our simulated sample datasets

| **True sepcies** | **Detected species** | **Original dataset** | **Deviating dataset** |
| --- | --- | --- | --- |
| *Bacillus anthracis* | *Bacillus bombysepticus* | 0 | 1 |
| *Bacillus anthracis* | *Bacillus cereus* | 0 | 3 |
| *Bacillus anthracis* | *Bacillus thuringiensis* | 0 | 6 |
| *Bacillus anthracis* | *Bacillus toyonensis* | 0 | 1 |
| *Bacillus anthracis* | *Bacillus weihenstephanensis* | 0 | 1 |
| *Bos taurus* | *Bos mutus* | 0 | 5 |
| *Bos taurus* | *Bubalus bubalis* | 1 | 7 |
| *Bos taurus* | *Muntiacus reevesi* | 0 | 1 |
| *Bos taurus* | *Ovis aries* | 0 | 2 |
| *Bos taurus* | *Pantholops hodgsonii* | 0 | 1 |
| *Bos taurus* | *Physeter catodon* | 0 | 1 |
| *Burkholderia mallei* | *Bacillus anthracis* | 52 | 48 |
| *Burkholderia mallei* | *Bacillus thuringiensis* | 10 | 11 |
| *Burkholderia mallei* | *Burkholderia pseudomallei* | 0 | 40 |
| *Clostridium botulinum* | *Bacillus anthracis* | 424 | 371 |
| *Clostridium botulinum* | *Bacillus cereus* | 8 | 0 |
| *Clostridium botulinum* | *Bacillus thuringiensis* | 0 | 2 |
| *Clostridium botulinum* | *Burkholderia mallei* | 3 | 2 |
| *Clostridium botulinum* | *Clostridium novyi* | 1 | 1 |
| *Clostridium botulinum* | *Staphylococcus aureus* | 0 | 1 |
| *Clostridium botulinum* | uncultured bacterium | 0 | 1 |
| *Escherichia coli* | *Acinetobacter baumannii* | 19 | 0 |
| *Escherichia coli* | *Bacillus cereus* | 100 | 0 |
| *Escherichia coli* | *Burkholderia mallei* | 80 | 64 |
| *Escherichia coli* | *Burkholderia pseudomallei* | 74 | 62 |
| *Escherichia coli* | *Klebsiella pneumoniae* | 0 | 9 |
| *Escherichia coli* | *Pseudomonas aeruginosa* | 41 | 36 |
| *Escherichia coli* | *Salmonella enterica* | 21 | 52 |
| *Staphylococcus aureus* | *Bacillus anthracis* | 246 | 207 |
| *Staphylococcus aureus* | *Bacillus cereus* | 20 | 0 |
| *Staphylococcus aureus* | *Bacillus thuringiensis* | 16 | 13 |
| *Staphylococcus aureus* | *Clostridium botulinum* | 0 | 1 |
| *Staphylococcus aureus* | *Staphylococcus capitis* | 0 | 2 |
| *Yersinia pestis* | *Bacillus anthracis* | 9 | 7 |
| *Yersinia pestis* | *Burkholderia mallei* | 19 | 13 |
| *Yersinia pestis* | *Burkholderia pseudomallei* | 11 | 11 |
| *Yersinia pestis* | *Yersinia pseudotuberculosis* | 7 | 35 |
| *Yersinia pestis* | *Yersinia similis* | 0 | 1 |

## Supplementary Table 2 - Comparison of RIEMS and MG-RAST results obtained for the simulated read dataset

The MG-RAST results were formatted to allow the comparison with the RIEMS results (assignments were limited to the best hit; annotation source set to ‘GenBank’ with a maximum e-value cut-off of 10^-4^; minimum percentage identity cut-off 60 %; minimum alignment length cut-off 15; result table grouped according to families). The columns “Deviation” hold information of the differences between the number of reads detected and the number of reads originally comprised. Note that the comparison in this table is based on results using databases as of the first quarter of 2013.

|  |  | **Assignments by** | | | |
| --- | --- | --- | --- | --- | --- |
|  |  | **MG-Rast normalised** | | **RIEMS** | |
| **Family** | **Reads actually comprised** | **Reads classified** | **Deviation** | **Reads classified** | **Deviation** |
|  |  |  |  |  |  |
| Bacillaceae | 12,774 | 12,581 | 193 | 11,856 | 918 |
| Burkholderiaceae | 16,154 | 18,304 | 2,150 | 16,433 | 279 |
| Clostridiaceae | 12,736 | 15,819 | 3,083 | 12,506 | 163 |
| Staphylococcaceae | 12,707 | 13,321 | 614 | 12,690 | 17 |
| Enterobacteriaceae | 25,221 | 25,496 | 275 | 25,232 | 11 |
| Paramyxoviridae | 418 | 387 | 31 | 418 | - |
| Bunyaviridae | 119 | 97 | 22 | 119 | - |
| Orthomyxoviridae | 1 | 1 | - | 1 | - |
| Bovidae | 4,909 | 592 | 4,317 | 4,907 | 2 |
| Canidae | 5,346 | - | 5,346 | 5,346 | - |
| Further families | - | 4,701 | 4,701 | 28 | 28 |
| Assigned | 90,385 | 91,299 | 914 | 89,536 | 849 |
| Unassigned | - | 2 |  | 849 |  |
| Total reads analysed | 90,385 | 91,301 |  | 90,385 |  |

## Supplementary Table 3 – Comparison of the run time (in min:sec) of the tools used on the different datasets

| **Dataset** | **RIEMS** | **Kraken** | **Clinical Pathoscope** | **Megablast vs. Metaphlan marker DB** |
| --- | --- | --- | --- | --- |
|  |  |  |  |  |
| RIEMS Original | 151:00 | 0:43 | 5:15 | 0:48 |
| RIEMS Deviating | 156:00 | 0:44 | 2:27 | 0:36 |
| Clinical PathoScope | 2783:00 | 8:06 | 22:09 | 43:19 |
